# Preliminary report of extracorporeal blood purification therapy in patients receiving LVAD: Cytosorb or Jafron HA330

**DOI:** 10.1051/ject/2023048

**Published:** 2024-03-15

**Authors:** Zhuldyz Nurmykhametova, Timur Lesbekov, Rymbay Kaliyev, Bolat Bekishev, Nilufar Jabayeva, Svetlana Novikova, Linar Faizov, Ivan Vakhrushev, Yuriy Pya

**Affiliations:** 1 Department of Perfusiology and Assisted Circulation Laboratory, National Research Cardiac Surgery Center Astana 010000 Kazakhstan; 2 Department of Adult Cardiac Surgery, National Research Cardiac Surgery Center Astana 010000 Kazakhstan; 3 Department of Anesthesiology and Intensive Care, National Research Cardiac Surgery Center Astana 010000 Kazakhstan

**Keywords:** Extracorporeal blood purification, Left ventricular assist devices, Cytokines

## Abstract

*Background*: Left ventricular assist device (LVAD) candidates are at increased risk of immune dysregulation and infectious complications. To attenuate the elevated proinflammatory cytokine levels and associated adverse clinical outcomes, it has been postulated that extracorporeal blood purification could improve the overall survival rate and morbidity of patients undergoing LVAD implantation. *Methods*: We retrospectively reviewed prospectively collected data of 15 patients who underwent LVAD implantation at our center between January 2021 and March 2022. Of these, 15 (100%) who received HeartMate 3™ (St. Jude Medical, Abbott, MN, USA) device were eligible. Intraoperatively, patients were single randomized 1:1:1 to three groups: *group* 1, patients who received Cytosorb therapy (*n* = 5; installed in the CPB circuit); *group* 2, patients who received Jafron HA330 (*n* = 5; installed in the CPB circuit); and control *group* 3, patients who did not receive filter (*n* = 5; usual care, neither Cytosorb nor Jafron during CPB). Baseline patient characteristics and intraoperative data were compared between the groups. Blood sample analyses were performed to assess the levels of inflammatory markers (IL-1, 6, 8; CRP, Leukocyte, Lactate, PCT, NT-proBNP, TNF-α) in both preoperative and postoperative data. *Results*: Baseline patient characteristics were similar in all three groups. We found that IL1α; IL 6; IL8; Lactatedehydrogenase, PCT, pro-BNP, CRP; Leukocyte, and TNFα levels significantly increased with LVAD implantation and that neither Cytosorb nor Jafron influenced this response. In-hospital mortality and overall survival during follow-up were similar among the groups. *Conclusion*: Our preliminary results showed that hemoadsorption therapy using Cytosorb or Jafron hemoadsorption (HA) 330 may not be clinically beneficial for patients with advanced heart failure undergoing LVAD implantation. Large prospective studies are needed to evaluate the potential role of HA therapy in improving outcomes in patients undergoing LVAD implantation.

## 1 Introduction

Left ventricular assist devices (LVADs) are a determined option for patients with end-stage heart failure refractory to standard medical therapy and are increasingly used either as a bridge to heart transplantation or as lifetime destination therapy [1]. However, owing to the presence of equipment, driveline exposure, surgical stress, and the inflammatory state associated with heart failure [2], LVAD candidates are at an increased risk of immune dysregulation and infectious complications [3]. In particular, elevation in plasma interleukin-6 (IL-6), interleukin-8 (IL-8), and tumor necrosis factor-α (TNFα) levels between pre- and post-surgery have been associated with worse post-implantation morbidity and mortality, and to a great extent, the development of multiorgan failure [4]. Therefore, to attenuate the elevated proinflammatory cytokine levels and associated adverse clinical outcomes, it has been postulated that extracorporeal blood purification could improve the overall survival rate and morbidity of patients undergoing LVAD implantation. Two similar hemoadsorption (HA) devices were used: Jafron HA 330 (HA 330, Jafron Biomedical Co., Ltd., China) and CytoSorb 300 (CytoSorb^®^, cartridge, Cytosorbents Europe GmbH, Germany) (see Table 1 for characteristics of the adsorbent cartridges).

**Table 1:** Characteristics of the CytoSorb300 and HA 330 cartridges.

Type of cartridges	CytoSorb 300 (Cytosorbents Europe GmbH)	HA 330(Jafron Biomedical Co., Ltd. China)
Adsorbent	Proprietary and patented cross-linked divinylbenzene polymer	A styrene-divinylbenzene copolymer
Cartridge volume (mL)	300	330
Adsorption spectrum	Small and mid-size hydrophobic molecules up to a size of approximately 60 kDa	With an approximate pore size distribution corresponding to a molecular weight range of 10–60 kDa
Maximum pressure limit	760 mm Hg	750 mm Hg
Maximum procedure duration	24 h	6 h
Anticoagulation	Heparin or citrate	Heparin or citrate
Priming fluid, procedure, and duration:	CytoSorb does not require priming/coating with heparin. Flushing with 2000 mL of 0.9% NaCl solution.	Flushing with 2500 ml of 0.9% NaCl solution + 12500ME heparin.
	Priming takes approximately 5 min.	The flushing takes about 50 min.

With this in mind, we hypothesize that Cytosorb or Jafron therapy benefits patients after LVAD implantation in terms of complications and overall survival.

## 2 Materials and methods

**Figure 1:**
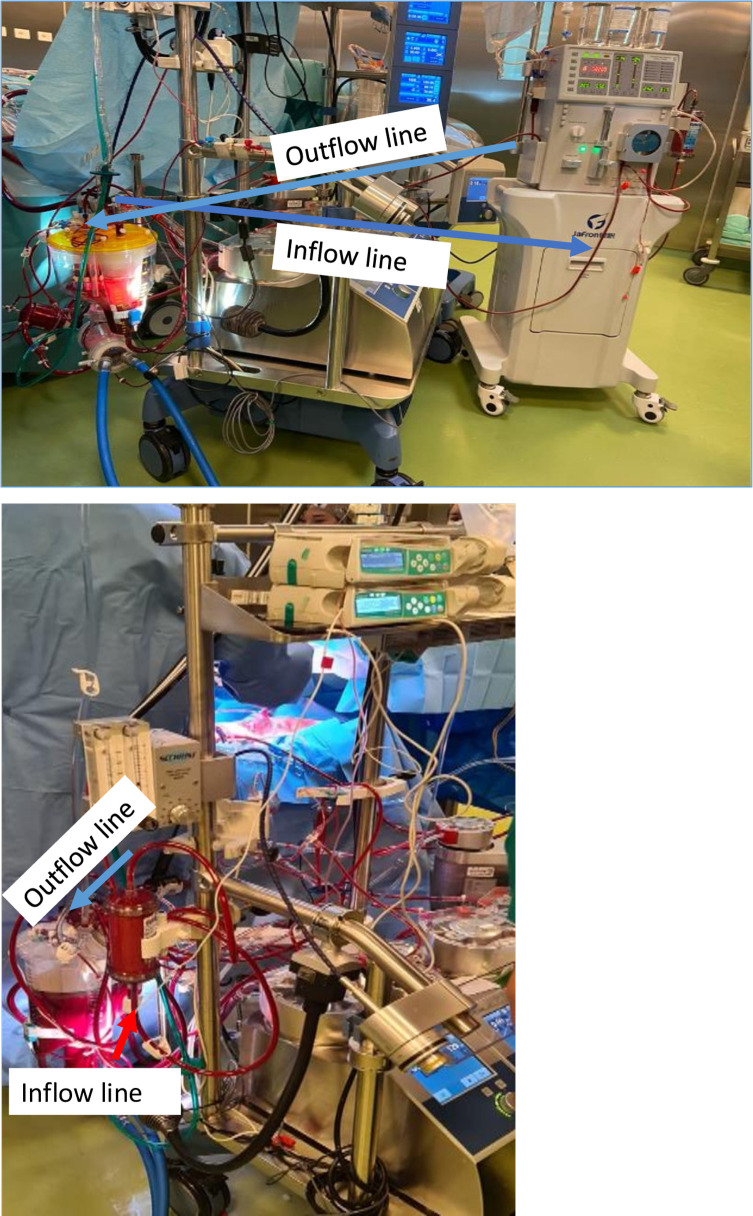
Picture of extracorporeal blood purification and CPB.

The study was approved by the Local Bioethics Committee of the National Research Cardiac Surgery Center (No. 01-74/2021 from 10/06/20) and registered in ClinicalTrials.gov PRS, Protocol registration and results system (NCT05090930). Two types of HA devices were used – Jafron HA 330 (HA 330, Jafron Biomedical Co., Ltd. China) and CytoSorb 300 (CytoSorb^®^, cartridge, Cytosorbents Europe GmbH, Germany) (see Table 1 for characteristics of the adsorbent cartridges). All patients provided written informed consent to participate in this study and to allow their data to be used for analysis. We retrospectively reviewed prospectively collected data from 15 patients who underwent LVAD HeartMate 3™ (St. Jude Medical, Abbott, MN, USA) device implantation at a single tertiary care center between January 2021 and March 2022. Intraoperatively, patients were single randomized 1:1:1 to three groups: *group* 1, patients who received Cytosorb therapy (*n* = 5; installed in the CPB circuit); *group* 2, patients who received Jafron HA330 (*n* = 5; installed in the CPB circuit); and control *group* 3, patients who did not receive filter (*n* = 5; usual care, neither Cytosorb nor Jafron during CPB. The filter was used at the discretion of the operator’s team based on the following clinical criteria: elevated inflammatory markers, hemodynamic instability requiring inotropic support before LVAD implantation, and redo LVAD implantation due to infection/redo after previous heart surgery. Preparatory washing of the adsorbents and heparinization during the procedures was carried out according to the manufacturer’s instructions. Anticoagulation was achieved by administering heparin (individual dosage, according to the laboratory data and the condition of the post-operative bleeding). All adsorption procedures were performed in a standard manner in HA 330 and Cytosorb groups. An extracorporeal blood purification filter was included via the side arm directly into the CPB circuit, and filtration was maintained over the entire CPB time. Intraoperative HA was performed using a standard CPB roller pump (Stockert S5, LivaNova Deutschland GmbH) with a mean aortic pressure of 68–84 mmHg and temperature control (35 °C). The HA 330 device was placed in the CPB venous circuit (Figure 1), using a haemoperfusion machine. The blood flow rate on the haemoperfusion machine was 160–200 mL/min. The Cytosorb cartridge was placed in the CPB without a haemoperfusion machine with an inflow arterial line and returned to the venous line. In all cases the cartridges after CPB are used by incorporating them into a hemodialysis machine or haemoperfusion machine (Jafron Company) if there is no need for RRT. The only difference was the manufacturer-defined duration of adsorption: 6 h for HA 330, and 24 h for Cytosorb.

Data were obtained from a prospectively collected institutional LVAD database that included detailed information on patient demographics, baseline clinical characteristics, laboratory and hemodynamic parameters, intraoperative variables, and postoperative outcomes. The patients were followed up for 30 days after hospital discharge.

Statistical analyses were performed using SPSS (version 26 IBM, USA). Chicago, IL, USA). Demographic and clinical baseline data are summarized as mean and standard deviation, expressed as minimum and maximum, for metric variables or absolute frequencies for categorical variables. All patients single randomized by 1:1:1 into three study groups Cytosorb, CS, Jafron HA, JHA, and Control, CO (neither Cytosorb nor Jafron during CPB). Differences between groups were analyzed using analysis of variance (ANOVA) to compare the means of two or more independent samples. A significant difference was assumed for *P*-values of < 0.05. The results are presented as medians with interquartile ranges.

## 3 Results

### 3.1 Baseline characteristics and intraoperative data

The baseline characteristics and intraoperative data are shown in Table 2. Blood samples were collected from all groups according to the scheme shown in Table 3. Baseline patient characteristics, such as sex and age, were similar in all three groups. Intraoperative data such as comorbidities, diagnosis, and INTERMACS profiles were comparable between the groups, as shown in Tables 2 and 4. There was no statistically significant difference in CBP time or cross-clamp time between the HA and control groups.

**Table 2:** Baseline characteristics of the study patients.

Characteristics		Cytosorb group (*n* = 5)	Jafron group (*n* = 5)	Control group (*n* = 5)	*p*
Demographic data	Age, years	50.4 ± 9.2	50.6 ± 11.2	50.8 ± 17.5	0.44
	Male, *n (%)*	5 (100%)	4 (80%)	3 (60%)	0.18
	BMI, kg/m^2^	26.8 ± 3.1	27.3 ± 2.6	26 ± 5.3	0.86
Comorbidities, *n (%)*	Coronary artery disease	2 (40%)	2 (40%)	1 (20%)	
	Atrial fibrillation	1 (10%)			
	Diabetes	2 (40%)	1 (20%)	3 (60%)	
	Stroke	2 (40%)	1 (20%)	2 (40%)	
	Infection	1 (20%)	2 (40%)	1 (20%)	
	Myocardial infarction	2 (40%)	2 (40%)	2 (40%)	
	Pulmonary hypertension	1 (20%)	2 (40%)	1 (20%)	
Primary diagnosis, *n (%)*	Ischemic cardiomyopathy	3 (60%)	1 (20%)	2 (40%)	
	Dilated cardiomyopathy	2 (40%)	4 (80%)	3 (60%)	
	Ejection fraction, %	16 (14–21)	15 (15–20)	17 (16–22)	
INTERMACS profile, *n* (%)	1–2	2 (40%)	3 (60%)	2 (40%)	
Device strategy at the time of implantation, *n* (%)	Destination therapy	5 (100%)	5 (100%)	5 (100%)	

Abbreviations: BMI, body mass index; INTERMACS, Interagency Registry for Mechanically Assisted Circulatory Support.

**Table 3:** Blood sampling scheme.

No. of blood sample	#1	#2	#3	#4	#5	#6	#7
HA group	Before HA/without HA therapy	At the beginning of CPB (initial 5 min)	At the beginning of HA therapy on CPB (initial 10 min after HA starting)	At the 60 min of HA therapy beginning on CPB	At the end of HA therapy on CPB	6 h after completion of the HA therapy	24 h after completion of the HA therapy

Abbreviations: HA, Hemoadsorption; CPB, cardiopulmonary bypass.

**Table 4:** Intraoperative data.

Characteristics		Cytosorb group (*n* = 5)	Jafron group (*n* = 5)	Control group (*n* = 5)	*p*
Duration, min	Operation	142.6 ± 26.4	132.6 ± 21.2	123.8 ± 17.5	0.42
	Cardiopulmonary bypass	102.6 ± 27.4	98.3 ± 20.5	92.2 ± 14.4	0.49
LVAD model, *n (%)*	HM 3	5 (100%)	5 (100%)	5 (100%)	
Isolated procedure, *n (%)*	LVAD implantation	2 (40%)	3 (60%)	4 (80%)	
Concomitant procedure, *n* (%)	Coronary artery bypass graft	2 (40%)	2 (40%)	1 (20%)	
	Mitral valve surgery	1 (20%)			

Abbreviation: LVAD, left ventricular assist device.

### 3.2 Laboratory parameters and adverse events

**Figure 2:**
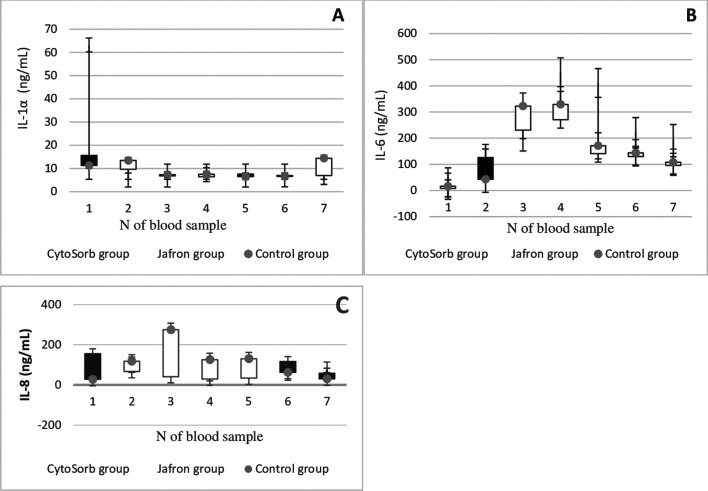
Laboratory trends of A – IL1α;interleukin1α; B – IL 6, interleukin 6; C – IL8, interleukin 8 in patients with LVAD Cytosorb, Jafron cartridge and in a cohort with LVAD alone. According to Table 1 blood sampling scheme.

**Figure 3:**
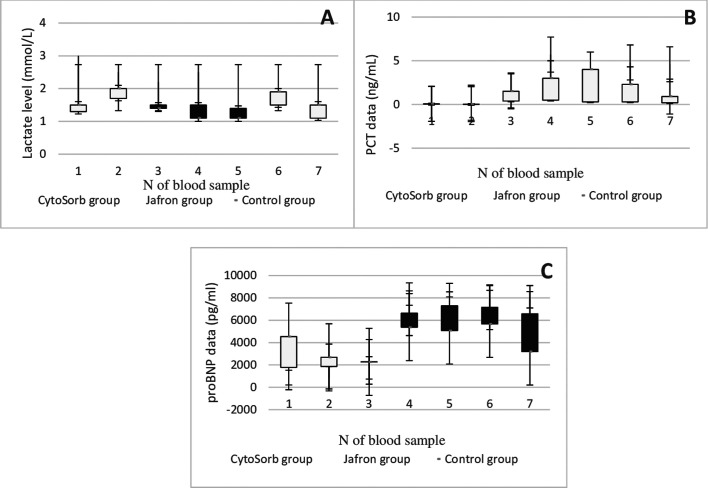
Laboratory trends of A – Lactate dehydrogenase; B – PCT, procalcitonin; C – proBNP, pro-b-type natriuretic peptide in patients with LVAD Cytosorb, Jafron cartridge and in a cohort with LVAD alone. According to Table 1 blood sampling scheme.

**Figure 4:**
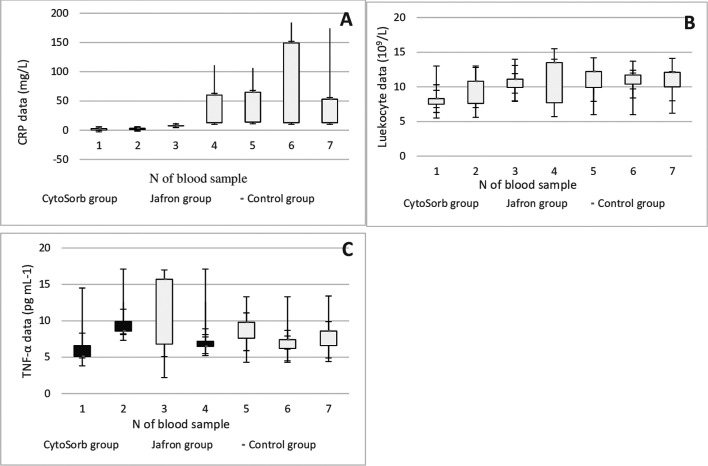
Laboratory trends of A – CRP, C-reactive protein; B –. Leukocyte; C – TNFα, tumor necrosis factor-alpha in patients with LVAD Cytosorb, Jafron cartridge and in a cohort with LVAD alone. According to Table 1 blood sampling scheme.

To evaluate the impact of cytokine adsorption on additional clinically relevant parameters, we determined interleukin (1α,6,8), Procalcitonin (PCT), N-Terminal Pro-B-type Natriuretic Peptide (NT-proBNP), C-reactive protein (CRP), Leukocyte, Tumor necrosis factor alpha (TNF-α) levels before and after the onset of HA as a measure of hemodynamic stabilization in all groups. Laboratory data of the Cytosorb, Jafron HA300, and control groups in pre-and postoperative values are presented in comparison shown in Figures 2–4A, 4B, 4C, respectively.

All the patients in the three groups had elevated levels of inflammatory markers in the perioperative and immediate postoperative periods. After 72 h of intensive care, the levels of blood inflammation markers tended to decline. No in-hospital mortality was observed. Over a median follow-up of 1 year between the groups, there were no survival differences (80%, 100%, and 80%). In both cases, the cause of death was multiorgan failure. The mean ICU stay duration (1, 2, and 2 days) and hospital stay duration (32, 29 and 24 days) were not significantly different between the Cytosorb, Jafron, and control groups. In-hospital major adverse events, such as acute kidney injury, the need for hemodialysis, and postoperative bleeding, were similar in all groups.

## 4 Discussion

Patients with advanced heart failure present with an increased inflammatory profile [2, 3]. The levels of proinflammatory cytokines in these patients are directly proportional to the severity of heart failure [5, 6]. Such a pre-existing inflammatory status in LVAD recipients, additional inflammation derived from surgical trauma and CPB, offers a clinical basis for the use of Cytosorb in this cohort [3, 7].

In this study, we analyzed the outcomes of patients with advanced heart failure who underwent LVAD implantation. We found that IL1α; IL 6; IL8; Lactatedehydrogenase, PCT, pro-BNP, CRP; Leukocyte, and TNFα levels significantly increased with LVAD implantation and that neither Cytosorb nor Jafron influenced this response. In-hospital mortality and overall survival during follow-up (30-day) were similar among the groups.

Zhigalov et al. [8] assessed the outcomes of 112 propensity score-matched patients with advanced heart failure who underwent LVAD. The authors found that WBC, CRP, and IL-6 significantly increased with LVAD implantation and that cytosorb did not influence this response. However, patients treated with Cytosorb developed respiratory failure, with mechanical ventilation for longer than 6 days post-implantation, and required tracheostomy during hospitalization. No complications were observed in our study. This explanation may be multifactorial. First, the number of patients included in this study was relatively low. The second patient in the two studies may have had different clinical characteristics. In addition, the duration of clinical implementation of HA devices were different. The latter discrepancy in our study was realized in the multiple points of analysis and prolonged usage of several cartridges. Knowing that inflammation is sometimes multifaceted and unpredictable, we studied the clearance of inflammatory markers, including in the control group. We focused on kinetics as the trend, as this method is more informative than an initial value and has never been studied before. Therefore, it is important to analyze the systemic inflammatory burden and disease dynamics. Moreover, as we treat critically ill patients, the clinical effects may not always be seen with or stable enough after the use of the first cartridge.

In summary, our preliminary results showed that HA using Cytosorb or Jafron HA 330 might not be clinically beneficial for LVAD recipients. Large prospective studies are needed to evaluate the potential role of HA therapy in improving outcomes in patients undergoing LVAD implantation.

### 4.1 Study limitations

Our study had some limitations. First, this study had a single-institution nature. Second, the number of patients included in this study was small, which limits the interpretation and generalization of our results. A missing anti-inflammatory phase will have a more deleterious effect than a high absolute proinflammatory value, followed by an equally high anti-inflammatory response.

## Data Availability

The data presented in this study are available upon request from the corresponding author.
